# Longitudinal trajectories of ADHD symptoms: relationships to early-childhood predictors and later outcomes

**DOI:** 10.64898/2026.10.05.26364348

**Published:** 2026-10-07

**Authors:** Emily Rounds, Michael Kozlowski, Shane McCormack, Peter Ryabinin, Elizabeth Nousen, Jessica Tipsord, Nathan F. Dieckmann, Sarah L. Karalunas, Megan M. Herting, Stephen V. Faraone, Molly Nikolas, Joel T. Nigg, Michael A. Mooney

## Abstract

**Background:**

ADHD symptoms follow heterogeneous trajectories across childhood and adolescence, with important implications for later functioning. Early-life factors, including perinatal health, family environment, cognition, socioeconomic conditions, and community exposures, may contribute to this variability. The present study examined (1) early-life predictors of ADHD symptom trajectories, and (2) ADHD symptom trajectories as predictors of later substance use, suicidality, and new-onset psychiatric disorders.

**Methods:**

Growth models were used to fit longitudinal trajectories of inattention, hyperactivity, and irritability measures in two independent samples: the Oregon ADHD-1000 cohort (N=1,215; 49.17% ADHD; 39.9% female, ages 7-23) and a subsample of the Adolescent Brain Cognitive Development (ABCD) study (N=1,252; 33.3% ADHD; 31.6% female, ages 9-16). Regression analyses examined multiple risk factors as predictors of symptom growth parameters as well as associations between symptom trajectories and later adverse outcomes.

**Results:**

In both cohorts, growth models fit well (CFI > .96, RMSEA < .04), with all symptoms decreasing over time ( *p* s < .001) and hyperactivity declining significantly faster than inattention. ADHD polygenic risk, family conflict, parenting, and cognition were associated with symptom intercepts in both cohorts ( *p* s < .001). In Oregon ADHD-1000, poorer baseline working memory and processing speed predicted slower declines in inattention ( *p* s < .001), but these effects did not replicate in ABCD; no other early-life factors predicted symptom trajectories. Increasing irritability trajectories predicted new disorder onset in both cohorts (ORs = 1.73–2.09, ps < .001), while more positive inattention slopes predicted new disorder onset in ABCD (OR = 3.07, p < .001) and showed a weaker association in Oregon ADHD-1000 (OR = 1.50, p = .007).

**Conclusion:**

While several early risk factors were associated with ADHD symptoms initially, none robustly predicted symptom trajectories over time. However, increasing irritability and inattention were robustly associated with increased risk for new disorder onsets later in adolescence.

**Key points:**

- ADHD symptoms decrease over time on average, with hyperactivity demonstrating the highest rate of decline.
- ADHD polygenic risk, family conflict, parental stress, parenting style, and cognition were strongly associated with symptom levels at intercept, but no replicable associations between early-life risk factors and change in symptoms over time (slopes) were observed.
- Irritability slope was robustly associated with new disorder onsets.

## Full Text Availability

The license terms selected by the author(s) for this preprint version do not permit archiving in PMC. The full text is available from the preprint server.

